# 4-(2-Meth­oxy­phen­yl)piperazin-1-ium 6-chloro-5-isopropyl-2,4-dioxopyrimidin-1-ide

**DOI:** 10.1107/S1600536814002256

**Published:** 2014-02-05

**Authors:** Fatmah A. M. Al-Omary, Hazem A. Ghabbour, Ali A. El-Emam, C. S. Chidan Kumar, Hoong-Kun Fun

**Affiliations:** a Department of Pharmaceutical Chemistry, College of Pharmacy, King Saud University, PO Box 2457, Riaydh 11451, Saudi Arabia; b X-ray Crystallography Unit, School of Physics, Universiti Sains Malaysia, 11800 USM, Penang, Malaysia

## Abstract

In the cation of the title salt, C_11_H_17_N_2_O^+^·C_7_H_8_ClN_2_O_2_^−^, the piperazine ring adopts a distorted chair conformation and contains a positively charged N atom with quaternary character. Its mean plane makes a dihedral angle of 42.36 (8)° with the phenyl ring of its 2-meth­oxy­phenyl substituent. The 2,4-dioxopyrimidin-1-ide anion is generated by deprotonation of the N atom at the 1-position of the pyrimidine­dione ring. Intra­molecular C—H⋯O hydrogen bonds generate *S*(6) ring motifs in both the cation and the anion. In the crystal, N—H⋯O, N—H⋯N and C—H⋯O hydrogen bonds are also observed, resulting in a two-dimensional network parallel to the *ab* plane. The crystal stability is further consolidated by weak C—H⋯π inter­actions.

## Related literature   

For the chemotherapeutic activity of pyrimidine-2,4-dione derivatives, see: Ghoshal & Jacob (1997 ▶); Spacilova *et al.* (2007 ▶); Blokhina *et al.* (1972 ▶); Tanaka *et al.* (1995 ▶); El-Emam *et al.* (2004 ▶); Al-Turkistani *et al.* (2011 ▶). For the acidity of pyrim­idine-2,4-dione derivatives, see: Kurinovich & Lee (2002 ▶); Jang *et al.* (2001 ▶); Nguyen *et al.* (1998 ▶). For the structures of other piperazinium salts, see: Craig *et al.* (2012 ▶); Dayananda *et al.* (2012 ▶); Fun *et al.* (2010 ▶). For reference bond lengths, see: Allen *et al.* (1987 ▶) and for hydrogen-bond motifs, see: Bernstein *et al.* (1995 ▶). For ring conformations and ring puckering analysis, see: Cremer & Pople (1975 ▶).
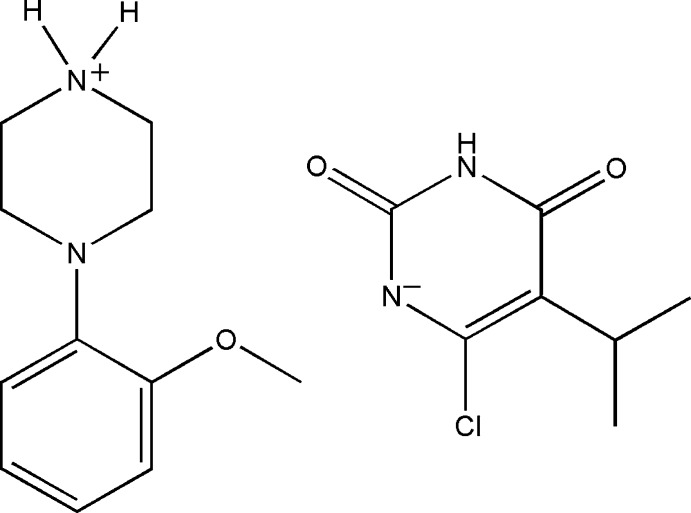


## Experimental   

### 

#### Crystal data  

C_11_H_17_N_2_O^+^·C_7_H_8_ClN_2_O_2_^−^
*M* *_r_* = 380.87
Monoclinic, 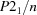

*a* = 8.9416 (2) Å
*b* = 10.5152 (3) Å
*c* = 20.5626 (5) Å
β = 98.832 (1)°
*V* = 1910.43 (8) Å^3^
*Z* = 4
Cu *K*α radiation
μ = 1.99 mm^−1^
*T* = 296 K
0.81 × 0.13 × 0.05 mm

#### Data collection  

Bruker APEXII CCD diffractometer
Absorption correction: multi-scan (*SADABS*; Bruker, 2009 ▶) *T*_min_ = 0.296, *T*_max_ = 0.907
11481 measured reflections
3531 independent reflections
3204 reflections with *I* > 2σ(*I*)
*R*_int_ = 0.033

#### Refinement  

*R*[*F* ^2^ > 2σ(*F* ^2^)] = 0.045
*wR*(*F* ^2^) = 0.118
*S* = 1.06
3531 reflections
251 parameters
H atoms treated by a mixture of independent and constrained refinement
Δρ_max_ = 0.33 e Å^−3^
Δρ_min_ = −0.32 e Å^−3^

### 

Data collection: *APEX2* (Bruker, 2009 ▶); cell refinement: *SAINT* (Bruker, 2009 ▶); data reduction: *SAINT*; program(s) used to solve structure: *SHELXTL* (Sheldrick, 2008 ▶); program(s) used to refine structure: *SHELXTL*; molecular graphics: *SHELXTL*; software used to prepare material for publication: *SHELXTL* and *PLATON* (Spek, 2009 ▶).

## Supplementary Material

Crystal structure: contains datablock(s) global, I. DOI: 10.1107/S1600536814002256/sj5388sup1.cif

Structure factors: contains datablock(s) I. DOI: 10.1107/S1600536814002256/sj5388Isup2.hkl

Supporting information file. DOI: 10.1107/S1600536814002256/sj5388Isup3.cml

CCDC reference:

Additional supporting information:  crystallographic information; 3D view; checkCIF report

## Figures and Tables

**Table 1:** Hydrogen-bond geometry (Å, °) *Cg*2 is centroid of the C1—C6 benzene ring.

*D*—H⋯*A*	*D*—H	H⋯*A*	*D*⋯*A*	*D*—H⋯*A*
N2—H2*N*2⋯O2^i^	0.892 (19)	1.881 (19)	2.7713 (18)	176 (2)
N2—H1*N*2⋯N4^ii^	0.92 (2)	1.987 (19)	2.8923 (19)	166.2 (18)
N3—H1*N*3⋯O2^iii^	0.87 (2)	2.02 (3)	2.8799 (18)	177 (2)
C8—H8*B*⋯O1	0.97	2.37	2.968 (2)	119
C9—H9*B*⋯O3^iv^	0.97	2.38	3.234 (2)	146
C17—H17*C*⋯O3	0.96	2.38	3.015 (3)	123
C10—H10*B*⋯*Cg*2^i^	0.97	2.65	3.4041 (17)	134

Symmetry codes: (i) 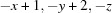; (ii) 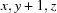; (iii) 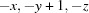; (iv) 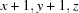.

**Fig. 1.**
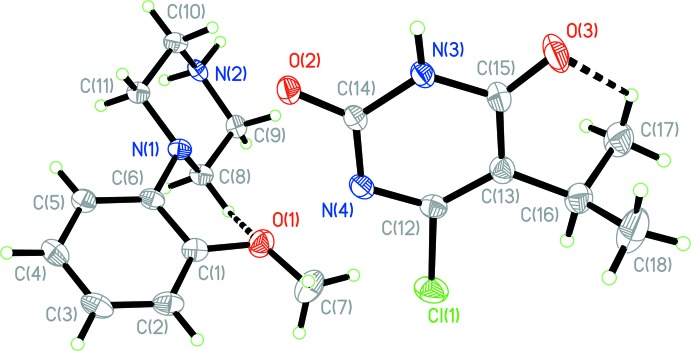
The molecular structure of the title compound with atom labels and 50% probability displacement ellipsoids. Intramolecular hydrogen bonds are drawn as dashed lines.

**Fig. 2.**
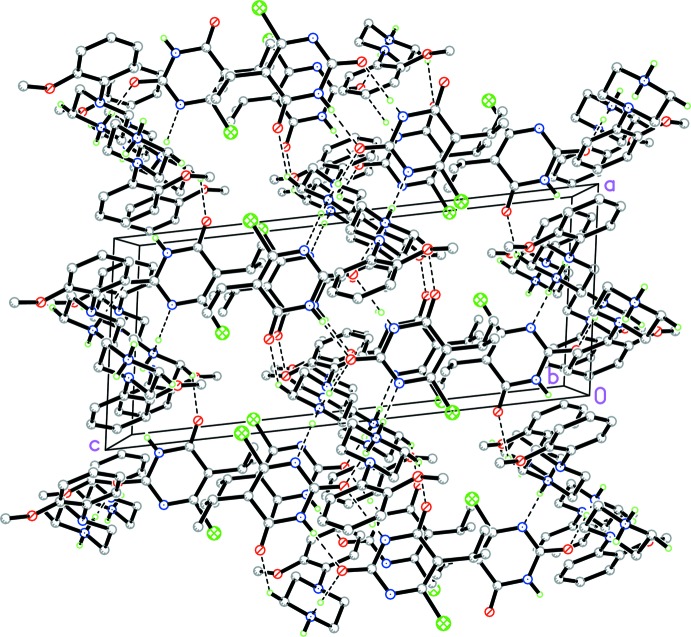
Crystal packing of the title compound, showing the hydrogen bonding interactions as dashed lines. H-atoms not involved in the hydrogen bonding are omited for clarity.

## supplementary crystallographic information

### 1. Comment

Pyrimidine-2,4-diones (uracils) and their derivatives have been known from much earlier times for their diverse chemotherapeutic properties including anticancer (Ghoshal & Jacob, 1997; Spacilova *et al.*, 2007; Blokhina *et al.*, 1972), anti-HIV (Tanaka *et al.*, 1995; El-Emam *et al.*, 2004) and antibacterial activities (Al-Turkistani *et al.*, 2011). The title piperazinium salt (I) was isolated as a minor by-product during the reaction of 6-chloro-5-isopropyluracil with 1-(2-methoxyphenyl)piperazine.

The asymmetric unit of (I) consists of a 4-(2-methoxyphenyl)piperazin-1-ium 6-chloro-5-isopropylpyrimidin-1-ide-2,4-dione cation-anion pair (Fig. 1). The 2,4-dioxopyrimidin-1-ide anion is generated by deprotonation of the N4 atom at the 1 position of the pyrimidine-dione ring (Kurinovich & Lee 2002; Jang *et al.*, 2001; Nguyen *et al.*, 1998). The six-membered piperazine ring (N1/C8/C9/N2/C10/C11) in the cation fragement adopts a slightly distorted chair conformation with puckering parameters: Q = 0.5774 (17) Å, θ = 177.86 (17) °, and φ = 129 (4) ° (Cremer & Pople, 1975) and contains a positively charged N atom (N2) with quaternary character. For an ideal chair configuration, θ has a value of 0 or 180°. The dihedral angle between the mean plane of the piperazine ring of the cation and the adjacent phenyl ring is 42.36 (8)°. Bond lengths (Allen *et al.*, 1987) and angles in the title compound are within normal ranges and are comparable with those reported earlier (Craig *et al.* 2012; Dayananda *et al.*, (2012); Fun *et al.*, 2010). Intramolecular C17–H17C···O3 and C8–H8B···O1 hydrogen bonds generate *S*(6) ring motifs in both the cation and anion (Fig 1), while a strong intermolecular N2–H1N2···N4_pyrimidine_ hydrogen bond links the two moieties. In the crystal, adjacent anionic species are interconnected *via* N2–H2N2···O2 and N3–H1N3···O2 hydrogen bonds (Table 1) with one bifurcated O acceptor atom on the anion resulting in *R*_2_^2^(9) and *R*_2_^2^(8) ring motifs (Bernstein *et al.*, 1995) respectively. The crystal structure features an intermolecular C9–H9B···O3 hydrogen bond (Fig. 2) which links the entities into a two-dimensional structure. The crystal packing is further stabilized by a weak intermolecular C10–H10B···*Cg*2^i^ interaction (Table 1) involving the centroid of the C1—C6 benzene ring.

### 2. Experimental

A mixture of 6-chloro-5-isopropyluracil (377 mg, 2.0 mmol), 1-(2-methoxyphenyl) piperazine (385 mg, 2.0 mmol) and anhydrous potassium carbonate (276 mg, 2.0 mmol), in ethanol (8 ml), was heated under reflux for 6 h. On cooling, the precipitate, thus formed was separated by filtration to yield 627 mg (91%) of 6-[4-(2-methoxyphenyl)-1-piperazinyl)]-5-isopropyluracil. The filtrate was concentrated by vacuum distillation to 5 ml and allowed to stand at room temperature overnight to yield 46 mg (6%) of the title salt (C_18_H_25_ClN_4_O_3_) as colourless plate-shaped crystals. M·P.: 517–519 K.

^1^H NMR (DMSO-d_6_, 500.13 MHz): δ 1.13 (d, 6H, CH_3_, J = 7.2 Hz), 2.53–2.56 (m, 1H, CH), 3.22–3.24 (m, 4H, Piperazine-H), 3.77 (s, 3H, OCH_3_), 3.42–3.45 (m, 4H, Piperazine-H), 6.78–7.02 (m, 4H, Ar—H), 8.02–8.14 (m, 2H, NH_2_), 10.88 (s, 1H, NH). ^13^C (DMSO-d_6_, 125.76 MHz): δ 19.50 (CH_3_), 26.90 (CH), 46.12, 49.86 (Piperazine-C), 56.80 (OCH3), 113.86, 119.12, 122.02, 122.98, 141.70, 148.28 (Ar—C), 123.90, 158.98, 162.82 (Pyrimidine-C),

### 3. Refinement

The nitrogen-bound H-atoms were located in a difference Fourier map and were refined freely. Other H atoms were positioned geometrically (C=H 0.93–0.98 Å) and refined using a riding model with *U*_iso_(H) = 1.2 *U*_eq_(C) or 1.5 *U*_eq_(C) for methyl H atoms. A rotating group model was used for the methyl group.

### Crystal data

**Table:**

C_11_H_17_N_2_O^+^·C_7_H_8_ClN_2_O_2_^−^	*F*(000) = 808
*M**_r_* = 380.87	*D*_x_ = 1.324 Mg m^−^^3^
Monoclinic, *P*2_1_/*n*	Cu *K*α radiation, λ = 1.54178 Å
Hall symbol: -P 2yn	Cell parameters from 3769 reflections
*a* = 8.9416 (2) Å	θ = 4.2–69.6°
*b* = 10.5152 (3) Å	µ = 1.99 mm^−^^1^
*c* = 20.5626 (5) Å	*T* = 296 K
β = 98.832 (1)°	Plate, colourless
*V* = 1910.43 (8) Å^3^	0.81 × 0.13 × 0.05 mm
*Z* = 4	

### Data collection

**Table:**

Bruker APEXII CCD diffractometer	3531 independent reflections
Radiation source: fine-focus sealed tube	3204 reflections with *I* > 2σ(*I*)
Graphite monochromator	*R*_int_ = 0.033
φ and ω scans	θ_max_ = 69.8°, θ_min_ = 4.4°
Absorption correction: multi-scan (*SADABS*; Bruker, 2009)	*h* = −10→10
*T*_min_ = 0.296, *T*_max_ = 0.907	*k* = −12→9
11481 measured reflections	*l* = −24→24

### Refinement

**Table:**

Refinement on *F*^2^	Secondary atom site location: difference Fourier map
Least-squares matrix: full	Hydrogen site location: inferred from neighbouring sites
*R*[*F*^2^ > 2σ(*F*^2^)] = 0.045	H atoms treated by a mixture of independent and constrained refinement
*wR*(*F*^2^) = 0.118	*w* = 1/[σ^2^(*F*_o_^2^) + (0.0615*P*)^2^ + 0.5789*P*] where *P* = (*F*_o_^2^ + 2*F*_c_^2^)/3
*S* = 1.06	(Δ/σ)_max_ = 0.001
3531 reflections	Δρ_max_ = 0.33 e Å^−^^3^
251 parameters	Δρ_min_ = −0.32 e Å^−^^3^
0 restraints	Extinction correction: *SHELXTL* (Sheldrick, 2008), Fc^*^=kFc[1+0.001xFc^2^λ^3^/sin(2θ)]^-1/4^
Primary atom site location: structure-invariant direct methods	Extinction coefficient: 0.0081 (5)

### Special details

**Table:**

Geometry. All e.s.d.'s (except the e.s.d. in the dihedral angle between two l.s. planes) are estimated using the full covariance matrix. The cell e.s.d.'s are taken into account individually in the estimation of e.s.d.'s in distances, angles and torsion angles; correlations between e.s.d.'s in cell parameters are only used when they are defined by crystal symmetry. An approximate (isotropic) treatment of cell e.s.d.'s is used for estimating e.s.d.'s involving l.s. planes.
Refinement. Refinement of *F*^2^ against ALL reflections. The weighted *R*-factor *wR* and goodness of fit *S* are based on *F*^2^, conventional *R*-factors *R* are based on *F*, with *F* set to zero for negative *F*^2^. The threshold expression of *F*^2^ > σ(*F*^2^) is used only for calculating *R*-factors(gt) *etc*. and is not relevant to the choice of reflections for refinement. *R*-factors based on *F*^2^ are statistically about twice as large as those based on *F*, and *R*- factors based on ALL data will be even larger.

### Fractional atomic coordinates and isotropic or equivalent isotropic displacement parameters (Å^2^)

**Table:**

	*x*	*y*	*z*	*U*_iso_*/*U*_eq_	
Cl1	0.50958 (5)	0.52933 (5)	0.21770 (2)	0.05543 (19)	
O2	0.20219 (13)	0.51968 (11)	0.00176 (5)	0.0368 (3)	
N4	0.34035 (14)	0.51859 (13)	0.10447 (7)	0.0340 (3)	
C14	0.20877 (17)	0.51323 (14)	0.06313 (7)	0.0300 (3)	
N3	0.07667 (15)	0.50064 (14)	0.08862 (7)	0.0347 (3)	
C15	0.0644 (2)	0.49113 (17)	0.15482 (8)	0.0387 (4)	
O3	−0.06114 (16)	0.47528 (17)	0.17059 (7)	0.0623 (4)	
C12	0.33057 (18)	0.51541 (15)	0.16938 (8)	0.0347 (3)	
C13	0.2058 (2)	0.50286 (16)	0.19945 (8)	0.0370 (4)	
C16	0.2061 (2)	0.49421 (19)	0.27315 (9)	0.0478 (4)	
H16A	0.3083	0.5163	0.2947	0.057*	
C17	0.0969 (3)	0.5878 (2)	0.29765 (10)	0.0591 (5)	
H17A	0.1235	0.6729	0.2871	0.089*	
H17B	0.1029	0.5796	0.3445	0.089*	
H17C	−0.0044	0.5697	0.2768	0.089*	
C18	0.1753 (4)	0.3582 (2)	0.29280 (12)	0.0783 (8)	
H18A	0.2574	0.3045	0.2849	0.118*	
H18B	0.0829	0.3287	0.2672	0.118*	
H18C	0.1661	0.3556	0.3387	0.118*	
O1	0.73479 (17)	0.92628 (13)	0.14090 (7)	0.0524 (3)	
N2	0.57634 (15)	1.36876 (13)	0.05996 (7)	0.0347 (3)	
N1	0.66629 (14)	1.10889 (13)	0.04804 (6)	0.0343 (3)	
C11	0.59845 (18)	1.18107 (15)	−0.00956 (8)	0.0359 (3)	
H11A	0.5429	1.1240	−0.0416	0.043*	
H11B	0.6774	1.2216	−0.0297	0.043*	
C5	0.79466 (19)	0.97928 (16)	−0.02689 (9)	0.0393 (4)	
H5A	0.7690	1.0388	−0.0602	0.047*	
C10	0.49247 (17)	1.28106 (16)	0.01019 (9)	0.0384 (4)	
H10A	0.4484	1.3291	−0.0283	0.046*	
H10B	0.4109	1.2403	0.0285	0.046*	
C8	0.75164 (18)	1.19407 (16)	0.09704 (8)	0.0375 (4)	
H8A	0.8336	1.2332	0.0784	0.045*	
H8B	0.7956	1.1449	0.1351	0.045*	
C3	0.9169 (2)	0.78466 (17)	0.00959 (10)	0.0485 (5)	
H3A	0.9744	0.7141	0.0018	0.058*	
C1	0.78835 (18)	0.90656 (16)	0.08317 (9)	0.0395 (4)	
C9	0.65130 (19)	1.29640 (16)	0.11833 (8)	0.0374 (4)	
H9A	0.5750	1.2581	0.1409	0.045*	
H9B	0.7114	1.3539	0.1487	0.045*	
C4	0.87617 (19)	0.87161 (18)	−0.03948 (10)	0.0457 (4)	
H4A	0.9028	0.8588	−0.0810	0.055*	
C6	0.75084 (17)	0.99994 (15)	0.03383 (8)	0.0344 (3)	
C2	0.8731 (2)	0.80127 (17)	0.07064 (10)	0.0473 (4)	
H2A	0.9005	0.7414	0.1036	0.057*	
C7	0.7453 (3)	0.8242 (2)	0.18654 (11)	0.0666 (6)	
H7A	0.6991	0.8485	0.2238	0.100*	
H7B	0.8499	0.8039	0.2008	0.100*	
H7C	0.6942	0.7511	0.1658	0.100*	
H2N2	0.646 (2)	1.408 (2)	0.0405 (10)	0.040 (5)*	
H1N2	0.511 (2)	1.428 (2)	0.0733 (10)	0.043 (5)*	
H1N3	−0.006 (3)	0.4964 (19)	0.0606 (11)	0.042 (5)*	

### Atomic displacement parameters (Å^2^)

**Table:**

	*U*^11^	*U*^22^	*U*^33^	*U*^12^	*U*^13^	*U*^23^
Cl1	0.0362 (3)	0.0756 (4)	0.0511 (3)	0.0075 (2)	−0.00393 (18)	−0.0107 (2)
O2	0.0322 (5)	0.0468 (7)	0.0329 (6)	−0.0035 (5)	0.0101 (4)	0.0021 (4)
N4	0.0269 (6)	0.0386 (7)	0.0378 (7)	0.0026 (5)	0.0087 (5)	−0.0008 (5)
C14	0.0288 (7)	0.0278 (7)	0.0351 (8)	0.0002 (5)	0.0105 (6)	0.0000 (5)
N3	0.0263 (7)	0.0453 (8)	0.0335 (7)	−0.0026 (5)	0.0077 (6)	−0.0013 (5)
C15	0.0376 (8)	0.0440 (9)	0.0371 (9)	−0.0034 (7)	0.0142 (7)	−0.0024 (7)
O3	0.0402 (7)	0.1034 (12)	0.0476 (8)	−0.0184 (7)	0.0209 (6)	−0.0075 (7)
C12	0.0319 (8)	0.0338 (8)	0.0379 (8)	0.0042 (6)	0.0038 (6)	−0.0034 (6)
C13	0.0396 (9)	0.0373 (8)	0.0353 (8)	0.0021 (6)	0.0098 (7)	−0.0021 (6)
C16	0.0551 (11)	0.0542 (11)	0.0350 (9)	0.0060 (9)	0.0101 (8)	−0.0027 (7)
C17	0.0753 (14)	0.0635 (13)	0.0417 (10)	0.0122 (11)	0.0194 (9)	−0.0062 (9)
C18	0.130 (2)	0.0601 (14)	0.0516 (12)	0.0141 (15)	0.0340 (14)	0.0112 (10)
O1	0.0662 (8)	0.0449 (7)	0.0478 (7)	0.0088 (6)	0.0147 (6)	0.0087 (6)
N2	0.0304 (6)	0.0304 (7)	0.0467 (8)	0.0003 (5)	0.0164 (6)	0.0005 (5)
N1	0.0326 (6)	0.0310 (7)	0.0383 (7)	0.0024 (5)	0.0029 (5)	−0.0018 (5)
C11	0.0347 (7)	0.0346 (8)	0.0379 (8)	0.0010 (6)	0.0037 (6)	−0.0005 (6)
C5	0.0348 (8)	0.0388 (9)	0.0442 (9)	−0.0001 (6)	0.0054 (7)	−0.0022 (7)
C10	0.0315 (8)	0.0360 (8)	0.0472 (9)	0.0024 (6)	0.0044 (7)	0.0029 (7)
C8	0.0348 (8)	0.0355 (8)	0.0410 (8)	0.0023 (6)	0.0019 (6)	−0.0025 (6)
C3	0.0374 (8)	0.0347 (9)	0.0734 (13)	0.0048 (7)	0.0090 (8)	−0.0090 (8)
C1	0.0364 (8)	0.0345 (8)	0.0464 (9)	−0.0008 (7)	0.0025 (7)	−0.0001 (7)
C9	0.0387 (8)	0.0359 (8)	0.0391 (8)	−0.0022 (7)	0.0107 (7)	−0.0021 (6)
C4	0.0378 (8)	0.0449 (10)	0.0558 (11)	0.0000 (7)	0.0112 (8)	−0.0123 (8)
C6	0.0278 (7)	0.0297 (8)	0.0449 (9)	−0.0005 (6)	0.0034 (6)	−0.0025 (6)
C2	0.0440 (9)	0.0333 (9)	0.0627 (11)	0.0052 (7)	0.0020 (8)	0.0044 (8)
C7	0.0879 (16)	0.0568 (13)	0.0546 (12)	−0.0033 (12)	0.0087 (11)	0.0155 (10)

### Geometric parameters (Å, º)

**Table:**

Cl1—C12	1.7557 (17)	N1—C6	1.427 (2)
O2—C14	1.2561 (19)	N1—C11	1.458 (2)
N4—C14	1.343 (2)	N1—C8	1.471 (2)
N4—C12	1.351 (2)	C11—C10	1.512 (2)
C14—N3	1.3702 (19)	C11—H11A	0.9700
N3—C15	1.386 (2)	C11—H11B	0.9700
N3—H1N3	0.86 (3)	C5—C6	1.382 (2)
C15—O3	1.226 (2)	C5—C4	1.392 (2)
C15—C13	1.449 (3)	C5—H5A	0.9300
C12—C13	1.362 (2)	C10—H10A	0.9700
C13—C16	1.518 (2)	C10—H10B	0.9700
C16—C18	1.523 (3)	C8—C9	1.508 (2)
C16—C17	1.526 (3)	C8—H8A	0.9700
C16—H16A	0.9800	C8—H8B	0.9700
C17—H17A	0.9600	C3—C4	1.369 (3)
C17—H17B	0.9600	C3—C2	1.383 (3)
C17—H17C	0.9600	C3—H3A	0.9300
C18—H18A	0.9600	C1—C2	1.388 (2)
C18—H18B	0.9600	C1—C6	1.415 (2)
C18—H18C	0.9600	C9—H9A	0.9700
O1—C1	1.362 (2)	C9—H9B	0.9700
O1—C7	1.419 (2)	C4—H4A	0.9300
N2—C9	1.491 (2)	C2—H2A	0.9300
N2—C10	1.491 (2)	C7—H7A	0.9600
N2—H2N2	0.89 (2)	C7—H7B	0.9600
N2—H1N2	0.93 (2)	C7—H7C	0.9600
			
C14—N4—C12	116.17 (13)	C10—C11—H11A	109.6
O2—C14—N4	122.35 (13)	N1—C11—H11B	109.6
O2—C14—N3	118.64 (14)	C10—C11—H11B	109.6
N4—C14—N3	119.00 (14)	H11A—C11—H11B	108.2
C14—N3—C15	125.85 (15)	C6—C5—C4	121.67 (17)
C14—N3—H1N3	116.7 (14)	C6—C5—H5A	119.2
C15—N3—H1N3	117.5 (14)	C4—C5—H5A	119.2
O3—C15—N3	118.90 (17)	N2—C10—C11	110.14 (13)
O3—C15—C13	126.11 (16)	N2—C10—H10A	109.6
N3—C15—C13	114.99 (14)	C11—C10—H10A	109.6
N4—C12—C13	129.23 (16)	N2—C10—H10B	109.6
N4—C12—Cl1	111.43 (12)	C11—C10—H10B	109.6
C13—C12—Cl1	119.34 (13)	H10A—C10—H10B	108.1
C12—C13—C15	114.63 (15)	N1—C8—C9	111.34 (13)
C12—C13—C16	125.66 (17)	N1—C8—H8A	109.4
C15—C13—C16	119.64 (15)	C9—C8—H8A	109.4
C13—C16—C18	110.40 (16)	N1—C8—H8B	109.4
C13—C16—C17	112.83 (16)	C9—C8—H8B	109.4
C18—C16—C17	111.53 (18)	H8A—C8—H8B	108.0
C13—C16—H16A	107.3	C4—C3—C2	120.27 (16)
C18—C16—H16A	107.3	C4—C3—H3A	119.9
C17—C16—H16A	107.3	C2—C3—H3A	119.9
C16—C17—H17A	109.5	O1—C1—C2	123.92 (16)
C16—C17—H17B	109.5	O1—C1—C6	116.29 (15)
H17A—C17—H17B	109.5	C2—C1—C6	119.78 (16)
C16—C17—H17C	109.5	N2—C9—C8	110.15 (13)
H17A—C17—H17C	109.5	N2—C9—H9A	109.6
H17B—C17—H17C	109.5	C8—C9—H9A	109.6
C16—C18—H18A	109.5	N2—C9—H9B	109.6
C16—C18—H18B	109.5	C8—C9—H9B	109.6
H18A—C18—H18B	109.5	H9A—C9—H9B	108.1
C16—C18—H18C	109.5	C3—C4—C5	119.60 (17)
H18A—C18—H18C	109.5	C3—C4—H4A	120.2
H18B—C18—H18C	109.5	C5—C4—H4A	120.2
C1—O1—C7	117.67 (16)	C5—C6—C1	118.03 (15)
C9—N2—C10	110.69 (13)	C5—C6—N1	122.93 (15)
C9—N2—H2N2	109.9 (13)	C1—C6—N1	119.01 (15)
C10—N2—H2N2	106.8 (13)	C3—C2—C1	120.56 (18)
C9—N2—H1N2	109.4 (13)	C3—C2—H2A	119.7
C10—N2—H1N2	110.2 (13)	C1—C2—H2A	119.7
H2N2—N2—H1N2	109.8 (18)	O1—C7—H7A	109.5
C6—N1—C11	114.77 (13)	O1—C7—H7B	109.5
C6—N1—C8	113.17 (12)	H7A—C7—H7B	109.5
C11—N1—C8	110.29 (13)	O1—C7—H7C	109.5
N1—C11—C10	110.09 (13)	H7A—C7—H7C	109.5
N1—C11—H11A	109.6	H7B—C7—H7C	109.5
			
C12—N4—C14—O2	177.57 (14)	N1—C11—C10—N2	58.83 (17)
C12—N4—C14—N3	−2.2 (2)	C6—N1—C8—C9	−171.18 (13)
O2—C14—N3—C15	179.40 (15)	C11—N1—C8—C9	58.72 (17)
N4—C14—N3—C15	−0.8 (2)	C7—O1—C1—C2	−10.5 (3)
C14—N3—C15—O3	−177.21 (17)	C7—O1—C1—C6	168.37 (18)
C14—N3—C15—C13	3.5 (2)	C10—N2—C9—C8	55.06 (16)
C14—N4—C12—C13	2.6 (2)	N1—C8—C9—N2	−56.08 (18)
C14—N4—C12—Cl1	−177.68 (11)	C2—C3—C4—C5	2.2 (3)
N4—C12—C13—C15	0.1 (3)	C6—C5—C4—C3	−1.3 (3)
Cl1—C12—C13—C15	−179.54 (12)	C4—C5—C6—C1	−1.4 (2)
N4—C12—C13—C16	177.04 (17)	C4—C5—C6—N1	−179.53 (15)
Cl1—C12—C13—C16	−2.6 (2)	O1—C1—C6—C5	−175.88 (15)
O3—C15—C13—C12	177.77 (18)	C2—C1—C6—C5	3.0 (2)
N3—C15—C13—C12	−3.0 (2)	O1—C1—C6—N1	2.4 (2)
O3—C15—C13—C16	0.7 (3)	C2—C1—C6—N1	−178.73 (15)
N3—C15—C13—C16	179.89 (15)	C11—N1—C6—C5	14.0 (2)
C12—C13—C16—C18	−105.4 (2)	C8—N1—C6—C5	−113.80 (17)
C15—C13—C16—C18	71.4 (2)	C11—N1—C6—C1	−164.14 (14)
C12—C13—C16—C17	129.0 (2)	C8—N1—C6—C1	68.06 (18)
C15—C13—C16—C17	−54.2 (2)	C4—C3—C2—C1	−0.5 (3)
C6—N1—C11—C10	171.16 (13)	O1—C1—C2—C3	176.69 (17)
C8—N1—C11—C10	−59.59 (16)	C6—C1—C2—C3	−2.1 (3)
C9—N2—C10—C11	−56.62 (16)		

### Hydrogen-bond geometry (Å, º)

Cg2 is centroid of the C1—C6 benzene ring.

**Table:**

*D*—H···*A*	*D*—H	H···*A*	*D*···*A*	*D*—H···*A*
N2—H2*N*2···O2^i^	0.892 (19)	1.881 (19)	2.7713 (18)	176 (2)
N2—H1*N*2···N4^ii^	0.92 (2)	1.987 (19)	2.8923 (19)	166.2 (18)
N3—H1*N*3···O2^iii^	0.87 (2)	2.02 (3)	2.8799 (18)	177 (2)
C8—H8*B*···O1	0.97	2.37	2.968 (2)	119
C9—H9*B*···O3^iv^	0.97	2.38	3.234 (2)	146
C17—H17*C*···O3	0.96	2.38	3.015 (3)	123
C10—H10*B*···*Cg*2^i^	0.97	2.65	3.4041 (17)	134

Symmetry codes: (i) −*x*+1, −*y*+2, −*z*; (ii) *x*, *y*+1, *z*; (iii) −*x*, −*y*+1, −*z*; (iv) *x*+1, *y*+1, *z*.
